# Prang's Chromos

**Published:** 1870-02

**Authors:** 


*Prang's Chromos.*—Good taste displayed in rendering Dental Rooms attractive and comfortable, is certain to be appreciated by all intelligent patients. All practitioners of Dentistry should be aware of this fact, and as paintings and engravings contribute greatly in improving the appearance of the office and reception rooms, they should be regarded as part of the necessary furniture.

We have before us one of Prang's American Chromos, entitled "Family Scene in Pompeii," from the painting of Joseph Coomans a Belgian artist of renown, who has devoted himself almost entirely to the delineation of the family life of Greek and Roman antiquity. The delicacy of finish and the brilliancy of coloring which this chromo-lithograph presents, shows it to be an admirable specimen of art.

The difference in price between these chromos and oil paintings of any merit, enables all to adorn their apartments at a comparatively small cost, with what are as attractive and beautiful as other more costly works of art.

"Corregio's Magdalena," "Sunset on the Coast," "Sunlight in Winter," "Esophus Creek," and "White Mountains" are other beautiful chromos among the collection published in Boston by L. Prang & Co.

